# Correlation Analysis between Uric Acid and Metabolic Syndrome in the Chinese Elderly Population: A Cross-Sectional Study

**DOI:** 10.1155/2023/8080578

**Published:** 2023-01-17

**Authors:** Guqiao Nie, Jing jing Wan, Lei Jiang, Shu kai Hou, Wen Peng

**Affiliations:** ^1^Department of General Practice, Union Hospital, Tongji Medical College, Huazhong University of Science and Technology, Wuhan, China; ^2^Community Health Service Center, Gutian Street, Qiaokou District, Wuhan, Hubei, China

## Abstract

**Background:**

Currently, both metabolic syndrome and hyperuricaemia have attracted extensive attention in public health. The correlation between uric acid and metabolic syndrome is controversial. Research on the relationship between uric acid and metabolic syndrome in community-dwelling elderly people is relatively lacking. The purpose of this study is to explore the relationship between uric acid and metabolic syndrome in the community-dwelling elderly people.

**Design:**

Cross-sectional study.

**Methods:**

We collected the physical examination data of 1,267 elderly people in Gutian community in Wuhan and used SPSS IBM 25.0 for data analysis. Correlation and logistic regression analyses were performed, and ROC curves were drawn.

**Results:**

The uric acid level of the nonmetabolic syndrome group was lower than that of the metabolic syndrome group (337.31 vs. 381.91 *µ*mol/L; *P* < 0.05). Uric acid was positively correlated with systolic blood pressure (*r* = 0.177, *P* < 0.001), diastolic blood pressure (*r* = 0.135, *P* < 0.001), body mass index (*r* = 0.234, *P* < 0.001), waist circumference (*r* = 0.283, *P* < 0.001), and triglycerides (*r* = 0.217, *P* < 0.05). High-density lipoprotein cholesterol (*r* = −0.268, *P* < 0.001) showed the opposite trend. Logistic regression analysis results suggested that uric acid is a risk factor for metabolic syndrome. The result is described as exp (B) and 95% CI (1.003 [1.001, 1.005]). Based on the receiver operating characteristic curve, we found that the area under the curve of uric acid to diagnose metabolic syndrome was 0.64 (sensitivity: 79.3%, specificity: 45.1%).

**Conclusion:**

We observed an association between uric acid levels and metabolic syndrome in the elderly Chinese population. The best threshold value for uric acid in predicting metabolic syndrome diagnosis was 314.5 *μ*mol/l.

## 1. Introduction

Metabolic syndrome (MetS) is a multifactorial pathological condition defined by the association of several metabolic disorders [1]. It is an independent risk factor for cardiovascular diseases (CVDs) and diabetes, which are often accompanied by increasing uric acid levels [2]. Experimental data have demonstrated that high uric acid is associated with endothelial dysfunction, oxidative stress, increased platelet adhesiveness and inflammation [3]. Numerous studies have shown a link between hyperuricaemia and other CVD risk factors, such as hypertension, obesity, dyslipidaemia, and diabetes [4–6]. The management of MetS and its definition in the elderly are particularly important. Whether uric acid has an effect on MetS is controversial [7].

Uric acid is an oxidation product of purine metabolism in the circulatory system. Studies have shown that high uric acid levels regulate the oxidative stress, inflammation, and enzymes associated with glucose and lipid metabolism, suggesting a mechanism for the impairment of metabolic homeostasis [8]. Hyperuricaemia can cause abnormal fat accumulation in body tissues, and adipokines can induce the production of reactive oxygen species and cause the formation of free radicals, which are pro-oxidant factors [9–12]. In contrast, uric acid is also considered to be an antioxidant that can fight superoxide anions, hydroxyl radicals, and peroxynitrite [7, 13]. Its antioxidant protection can be reflected in cell apoptosis, cancer, and ageing [14]. Oxidative stress is inseparable from the formation of MetS [15]. The two-way regulation of oxidative stress by uric acid might be closely related to the level of uric acid in people with MetS. Therefore, we designed a cross-sectional clinical study to explore the correlation between the two.

Our research focused on the level of uric acid and MetS in the elderly and explored the relationship between them. The optimal cut-off value of uric acid at risk of MetS was initially obtained.

## 2. Materials and Methods

### 2.1. Data and Participants

This was an observational study of 1,267 elderly people (556 men and 711 women) who had health examinations at a community medical examination centre between January 1, 2016 and December 31, 2016. We collected consecutive participant information over a period of time. Inclusion criteria (all of the following): (1) Asian; (2) ≥65 years old; and (3) complete information attainable. Exclusion criteria (at least one of the following): (1) acute infection period; (2) disabled person; (3) hormone therapy; (4) diagnosis of malignant tumour; and (5) acute cardiovascular and cerebrovascular disease.

### 2.2. Definition of MetS

Diagnosis of MetS according to the Chinese Medical Association Diabetes Society Metabolic Syndrome Diagnosis and Treatment Guidelines (CDS): (1) body mass index (BMI) ≥ 25 m/kg^2^; (2) triglyceride (TG) ≥ 1.7 mmol/l; (3) HDL-C < 0.9 mmol/l (male)  < 1.0 mmol/l (female); (4) systolic blood pressure and diastolic blood pressure (SBP/DBP) ≥ 140/90 mmHg and/or with diagnosed and treated hypertension; and (5) fasting blood glucose (FBG) ≥ 6.1 mmol/l, 2 h postprandial blood glucose (PBG) ≥ 7.8 mmol/l, or diagnosed with diabetes. MetS is diagnosed by three or more of the above items.

### 2.3. Definition of Hyperuricaemia

The diagnostic criteria for hyperuricaemia were based on the “China Guidelines for Diagnosis and Treatment of Hyperuricaemia and Ventilation 2019”. Uric acid levels above 420 µmol/L are defined as hyperuricemia.

### 2.4. General Data and Biochemical Indicators

Data for this study were obtained from the physical examination records of elderly people in the community. For example, age and sex were recorded orally in the physical examination information by the participant. Height was measured in a standing position without shoes using a stadiometer with a sensitivity of 0.1 cm. Weight was measured wearing light clothing using a scale sensitive to the nearest 0.1 kg. BMI was calculated by dividing the person's weight (kg) by their height squared (m^2^). Waist circumference (WC) was measured using plastic tape to the nearest 0.1 cm. The doctor used a mercury sphygmomanometer on the upper arm to measure blood pressure, systolic blood pressure and diastolic blood pressure. The average value of two blood pressure measurements taken at least five minutes apart was used. Biochemical indices, including FBG, blood lipids, and uric acid, were all measured using venous blood obtained from the participants on an empty stomach. Levels of serum UA, TGs, HDL-C, low-density lipoprotein cholesterol (LDL-C), and FBG were measured using Roche E602 and Roche C701 (both of which are automatic biochemical analysers).

### 2.5. Statistical Analysis

IBM SPSS version 25.0 statistical software was used for data analysis in this study. The normality of distributions was evaluated using the Kolmogorov–Smirnov test, and continuous data are presented as the mean ± SD. Categorical variables are presented as the frequency (percentage). The statistical significance of the differences in clinical and biochemical values between participants with and without MetS was analysed using Student's *t*-test for continuous variables and the chi-squared test for categorical variables. Kendall correlation analysis was used to analyse the correlation between two categorical variables, Pearson correlation analysis was used to examine the relationship between two continuous variables, and Spearman correlation analysis was used to investigate the relationship between categorical variables and continuous variables. After drawing the receiver operating characteristic (ROC) curve, the cut-off value of the uric acid level was obtained by calculating the Youden index (sensitivity + specificity − 1). The uric acid level corresponding to the maximum value of the Youden index is the optimal cut-off value. Considering the effect of gender in metabolic syndrome, we also contended to plot ROC curves separately for the male and female elderly population.

## 3. Results

### 3.1. Characteristics of the Research Population

The average age of the population was 71.64 ± 5.61 years. The basic laboratory and clinical characteristics of 1,267 participants (556 men and 711 women) with and without MetS enrolled in this study are shown in Table 1. The prevalence of hyperuricaemia and MetS was 28.1% (356 of 1,267) and 18.6% (128 of 1,267), respectively. The prevalence of MetS in the hyperuricaemia population was 28.4% vs. 14.6% in the nonhyperuricaemia population. In the group diagnosed with MetS, age, WC, blood pressure, TG, TC, LDL-C, UA, and FBG were significantly higher than those in the non-MetS group, and there were significant differences (*P* < 0.05). The level of HDL-C in the MetS group was significantly lower than that in the non-MetS group (*P* < 0.05). In addition, the average serum uric acid level was higher than that in subjects without MetS, and this difference was significant (381.91 vs. 337.31 *µ*mol/L; *P* < 0.05), as shown in Table 1.

### 3.2. Correlation Analysis

We performed simple correlation analysis to explain the relationship between uric acid levels and various components of MetS. We also performed a simple correlation analysis with MetS as the dependent variable (shown in Table 2). The results were expressed by the correlation coefficient *r* and *P* value. We found that WC (*r* = 0.283, *P* < 0.001), BMI (*r* = 0.234, *P* < 0.001), and TG (*r* = 0.217, *P* < 0.05) were positively correlated with uric acid levels, and high-density lipoprotein (*r* = −0.268, *P* < 0.001) was significantly negatively correlated with uric acid levels. In addition, we used metabolic syndrome as the dependent variable to perform a simple correlation analysis. The correlation analysis results are shown in the right column of Table 2.

### 3.3. Logistic Regression between Uric Acid Levels and MetS

In the logistic regressions, using MetS as the dependent variable, we identified that uric acid was a risk factor for MetS. We included the relevant variables in the regression model to construct three regression models, as shown in Table 3. The basis for the inclusion of the regression model was univariate correlation variables obtained from the analysis as well as clinically known and recognized variables associated with MetS. Model 1 is a rough model without any adjustment for confounding factors, Model 3 is a fully corrected model, and Model 2 is a partially corrected model. The results are expressed as exp (B) (OR value) and 95% CI. In Model 3, the confounding of factors, such as age, sex, WC, blood pressure, blood lipids, and FBG, were adjusted, and the results showed that uric acid level might be an independent risk factor for MetS (exp (B) = 1.003, 95% CI (1.001, 1.005), *P*=0.014). Obesity (obesity defined as BMI ≥ 25 kg/m^2^) is an important part of the metabolic syndrome, and both the simple correlation studies and regression results described above suggest that BMI and uric acid levels are closely related (Table 2). Therefore, we performed a stratified analysis according to whether or not they were obese. We could see an association between uric acid and metabolic syndrome in both obese and nonobese populations (ORs 1.002, 1.006, *P* values <0.05, respectively), independent of obesity (Table 4).

### 3.4. ROC Curves between Uric Acid Level and MetS

**Figure 1:**
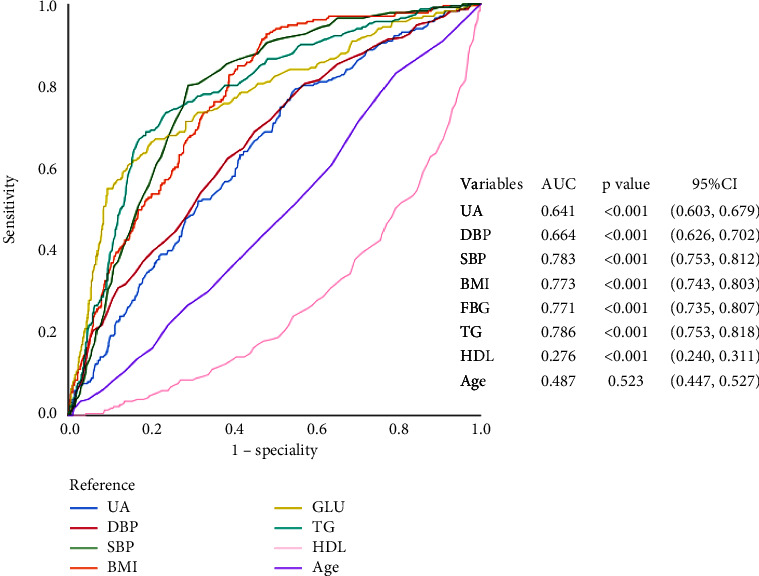
ROC curves of uric acid to predict the presence of MetS in all participants.

**Figure 2:**
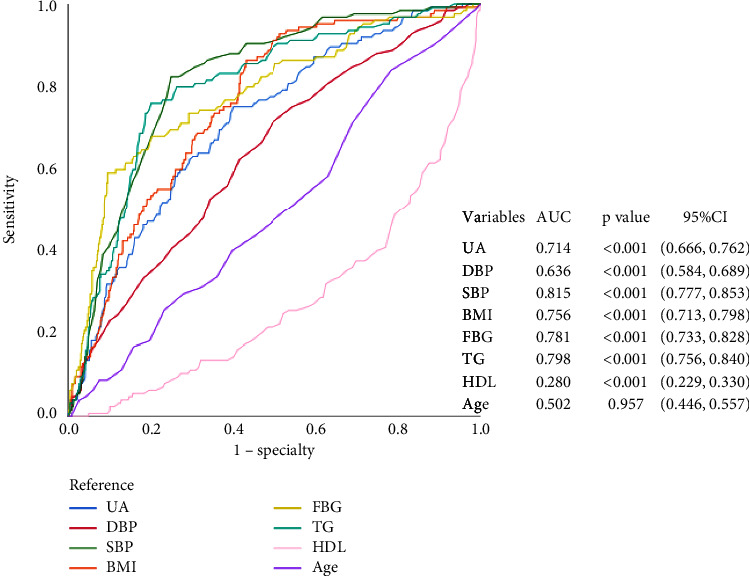
ROC curves of uric acid to predict the presence of MetS in female.

**Figure 3:**
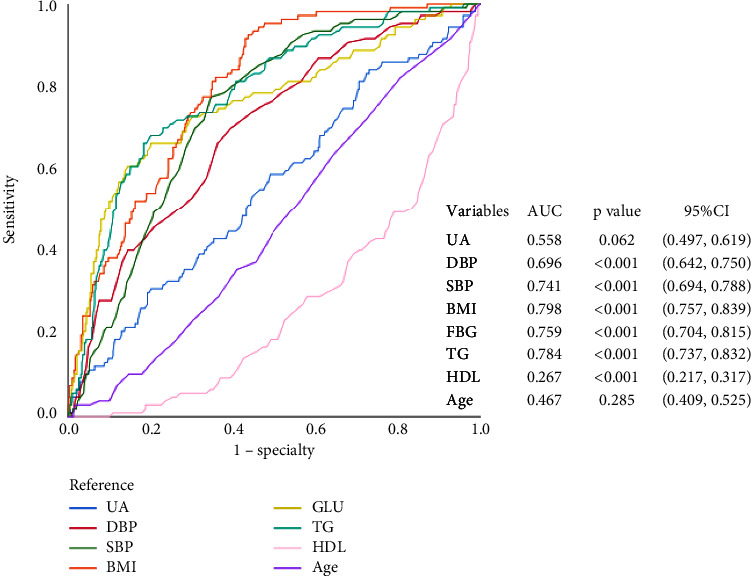
ROC curves of uric acid to predict the presence of MetS in male.

To predict a threshold value for the diagnosis of MetS using uric acid, ROC curves were drawn.  Figure 1 shows the ROC curves for the diagnosis of MetS by different variables. The AUC of UA, TG, TC, GLU, HDL, LDL, BMI, and age were 0.641, 0.786, 0.542, 0.771, 0.276, 0.544, 0.773, and 0.487, respectively. By calculating the Youden index, we found that the cut-off value for uric acid corresponding to the maximum Youden index (0.244) was 314.5 *μ*mol/l. The Youden index corresponded to a sensitivity and specificity of 79.3% and 45.1%, respectively. In this community population, the prevalence of metabolic syndrome was 19.24% and 17.86% in elderly males and elderly females, respectively. Considering the influence of gender in this, we plotted ROC curves in the elderly male and female populations, respectively, and we found different results (Figures 2 and 3). The diagnostic efficacy of uric acid for metabolic syndrome seems to be more significant in the elderly male population, with an area under the curve of 0.735 and a sensitivity and specificity of 75.2% and 60%, respectively. And the cut-off value for uric acid is 314.5 *μ*mol/l too.

## 4. Discussion

Global ageing is becoming increasingly serious, and achieving healthy “ageing” can reduce the burden on national finances and improve the quality of life of the elderly. According to the data of the World Health Organization, the prevalence of MetS in the elderly ranges from 11% to 43% (median 21%) and NCEP ranges 23% to 55% (median 31%) [16–18]. We conducted a study in a community-based elderly population and found that the uric acid level might be independently associated with the risk of MetS and determined that a uric acid level of 314.5 *μ*mol/l was the optimal cut-off value.

In fact, there are different opinions about the correlation between uric acid and MetS. An increase of 65% in the risk of MetS per standard deviation increase in uric acid was found using unadjusted observational analyses. This association attenuated upon adjustment for potential confounders. Mendelian randomization analyses showed no evidence of a causal association between uric acid and MetS and MetS components [19]. Adnan et al. investigated 102 outpatients. In subjects with MetS, the average serum uric acid level was higher than that in subjects without MetS, but this difference was not significant (6.62 vs. 6.28 mg/dL; *P*=0.556) [20]. However, some studies even showed that uric acid is an independent risk factor for MetS [21, 22]. Cibičková et al. conducted an investigation and found that uric acid is related to MetS, and the correlation is most obvious in the case of dyslipidaemia. Markers of lipid metabolism showed moderate correlations (correlation coefficient *r* = 0.4 – 0.6) with uric acid levels (positive correlation with TAG and AIP and negative correlation with HDL cholesterol), whereas parameters of insulin resistance (glycaemia, insulin, C-peptide, and HOMA-IR) showed low positive correlations (correlation coefficient *r* = 0.1 – 0.3) with uric acid levels [23]. The above conclusions are consistent with our findings.

A 5-year retrospective cohort study of healthy Japanese adults reported that elevated SUA increased the risk of developing high LDL cholesterol as well as hypertriglyceridemia [24]. In this cross-sectional study, we came to similar conclusions with a significant correlation between uric acid levels and blood lipids (triglycerides and HDL-C). A study suggested that higher intracellular uric acid levels can induce mitochondrial translocation of the nicotinamide adenine dinucleotide phosphate oxidase subunit nicotinamide adenine dinucleotide phosphate oxidase 4, further leading to increased mitochondrial oxidative stress, mitochondrial dysfunction, and citrate release to the cytosol, ultimately promoting the synthesis of lipids and TG [25]. In addition, both soluble and crystalline uric acid inhibit AMP-kinase, leading to reduced fatty acid oxidation and triglyceride accumulation [26]. Hyperinsulinaemia in the body increases the reabsorption of uric acid in the renal tubules, thereby forming hyperuricaemia. In addition, the enzyme activity that catalyses the decomposition of TG is affected by high levels of uric acid, which inhibits the decomposition of serum TG, leading to the incidence of hypertriglyceridaemia [27].

Experimental studies have shown that hyperuricaemia may mediate insulin resistance in models of fructose-dependent and fructose-independent metabolic syndrome [7, 28]. MetS is defined as IR syndrome, which can lead to the occurrence of MetS [29]. Studies have suggested that hyperuricaemia and IR have a bidirectional relationship [30]. Increasing serum uric acid can lead to IR through the low-pressure bioavailability of nitric oxide (NO) and ultimately produce oxidative stress in the mitochondria [20]. IR can also cause hyperuricaemia by increasing the sodium reabsorption mechanism and increasing the absorption of uric acid. The increase in serum uric acid is negatively correlated with insulin sensitivity [31]. In people with hyperuricaemia, the use of allopurinol or benzbromarone may improve insulin resistance in patients with the metabolic syndrome [32]. Overall, insulin resistance may be the mechanism explaining the association between uric acid and metabolic syndrome. Women with metabolic syndrome and general obesity are at higher risk of developing severe hyperuricaemia compared to men [33]. In a large prospective cohort study of middle-aged and elderly Chinese, researchers also found that the association between uric acid and triglycerides was more pronounced in women [34]. When we plotted the ROC curves, we found differences in the diagnostic efficacy of uric acid for metabolic syndrome by gender, which seemed to be more significant in the female population.

Several limitations of this study should be mentioned. First, cross-sectional research cannot draw a causal relationship between MetS and uric acid affected by the type of research, and the degree of risk cannot be measured in this study design. Second, we could not obtain some useful data, such as information on drug use. Therefore, we hope to conduct multicentre and prospective research in the future. In summary, we hope to bring new content to the management of MetS for the elderly in China through our research. The common pathogenesis of hyperuricaemia and MetS is also worthy of further exploration.

## Figures and Tables

**Table 1:** Clinical characteristics of different groups.

Characteristic or parameter	MetS (*N* = 234)	Non-MetS (*N* = 1033)	*P* value
	Mean ± SD	Mean ± SD	

Age (years)	71.64 ± 5.605	71.39 ± 5.409	<0.001
WC (cm)	93.87 ± 8.407	86.51 ± 8.91	<0.001
BMI (kg/m^2^)	27.14 ± 3.19	23.98 ± 3.25	<0.001
DBP (mm·Hg)	82.97 ± 10.97	76.54 ± 11.29	<0.001
SBP (mm·Hg)	148.08 ± 13.82	131.26 ± 18.17	<0.001
TG (mmol/L)	2.02 ± 0.96	1.31 ± 0.75	<0.001
TC (mmol/L)	4.78 ± 1.10	4.60 ± 0.89	0.024
HDL (mmol/L)	1.02 ± 0.22	2.75 ± 0.77	<0.001
LDL (mmol/L)	2.92 ± 0.91	2.76 ± 0.76	0.005
UA (*µ*mol/L)	381.91 ± 95.01	337.31 ± 89.48	<0.001
FBG (mmol/L)	6.76 ± 2.03	5.33 ± 1.18	<0.001

	*N* (%)	*N* (%)	
Sex (% male)	107 (45.72)	449 (43.46)	0.56
Hyperuricaemia (% yes)	101 (43.16)	255 (24.68)	<0.001

**Table 2:** Bivariate correlation analysis.

Variables and uric acid			Variables and MetS		
Variable	*r*	*P* value	Variable	*r*	*P* value
Sex	0.350^*∗∗*^	<0.001	Sex	0.018	0.53
Age (years)	0.114^*∗∗*^	<0.001	Age	−0.012	0.672
SBP (mm Hg)	0.177^*∗∗*^	<0.001	BMI	0.363^*∗∗*^	<0.001
DBP (mm Hg)	0.135^*∗∗*^	<0.001	DBP	0.217^*∗∗*^	<0.001
BMI (kg/m^2^)	0.234^*∗∗*^	<0.001	SBP	0.381^*∗∗*^	<0.001
Waist (cm)	0.283^*∗∗*^	<0.001	TG	0.383^*∗∗*^	<0.001
FBG (mmol/L)	0.047	0.094	Waist	0.308^*∗∗*^	<0.001
TG (mmol/L)	0.217^*∗∗*^	<0.05	TC	0.057^*∗*^	0.044
TC (mmol/L)	−0.051	0.071	HDL	−0.301^*∗∗*^	<0.001
HDL (mmol/L)	−0.268^*∗∗*^	<0.001	LDL	0.059^*∗*^	<0.001
LDL (mmol/L)	0.007	0.803	UA	0.189^*∗∗*^	<0.001
			FBG	0.364^*∗∗*^	<0.001

**Table 3:** Logistic regression results of the association of participant characteristics with MetS.

Exposure	Model 1			Model 2			Model 3		
	B	Exp (B) (95% CI)	*P*value	B	Exp (B) (95% CI)	*P*value	B	Exp (B) (95% CI)	*P*value
UA (*μ*mol/l)	0.005	1.005 (1.003, 1.006)	<0.001	0.003	1.003 (1.001, 1.005)	<0.001	0.003	1.003 (1.001, 1.005)	0.011
Sex (male)	—	—	—	0.153	1.165 (0.791, 1.627)	0.421	0.15	1.161 (0.724, 1.863)	0.535
Age (years)	—	—	—	−0.033	0.968 (0.938, 0.999)	0.041	−0.03	0.971 (0.935, 1.007)	0.117
SBP (mm Hg)	—	—	—	0.053	1.054 (1.043, 1.065)	<0.001	0.068	1.07 (1.056, 1.084)	<0.001
BMI (kg/m^2^)	—	—	—	0.236	1.267 (1.156, 1.387)	<0.001	0.331	1.393 (1.248, 1.555)	<0.001
WC (cm)	—	—	—	0.01	1.01 (0.977, 1.044)	0.569	−0.034	0.966 (0.929, 1.006)	0.095
FBG (mmol/L)	—	—	—	—	—	—	0.605	1.831 (1.611, 2.081)	<0.001
TG (mmol/L)	—	—	—	—	—	—	0.705	2.024 (1.568, 2.582)	<0.001
HDL (mmol/L)	—	—	—	—	—	—	−3.16	0.042 (0.015, 0.12)	<0.001

**Table 4:** Association between uric and MetS in obesity and nonobesity population.

Obesity	Model 1			Model 2		
	Exp (B)	95%CI	*P* value	Exp (B)	95%CI	*P* value
Yes	1.005	1.002–1.008	0.001	1.002	1.000–1.005	0.049
No	1.003	1.001–1.005	<0.001	1.006	1.002–1.010	0.004

Model 1: crude model. Model 2: adjusted for age, gender, waist, blood pressure, triglycerides, and HDL-C.

## Data Availability

The data used for this paper are available from the corresponding author upon reasonable request.

## Abbreviations

MetS: Metabolic syndrome
BMI: Body mass index
BP: Blood pressure
CVD: Cardiovascular disease
FBG: Fasting blood glucose
HDL-C: High-density lipoprotein cholesterol
LDL-C: Low-density lipoprotein cholesterol
TC: Total cholesterol
TG: Triglyceride
UA: Uric acid
WC: Waist circumstance.
